# A Theoretical Twist on the Transparency of Open Notes: Qualitative Analysis of Health Care Professionals’ Free-Text Answers

**DOI:** 10.2196/14347

**Published:** 2019-09-25

**Authors:** Gudbjörg Erlingsdóttir, Lena Petersson, Karin Jonnergård

**Affiliations:** 1 Department of Design Sciences Lund University Lund Sweden; 2 Department of Business Administration Lund University Lund Sweden

**Keywords:** electronic health record, eHealth, transparency, mental health, Open Notes, psychiatry, health care professionals, Web survey, free-text answers

## Abstract

**Background:**

The New Public Management movement strove for transparency so that policy makers and citizens could gain insight into the work and performance of health care. As the use of the electronic health record (EHR) started to diffuse, a foundation was laid for enhanced transparency within and between health care organizations. Now we appear to be experiencing a new kind of transparency in the health care sector. Many health care providers offer their patients online access to their EHRs (here referred to as Open Notes). The Open Notes system enables and strives for transparency between the health care organization and the patient. Hence, this study investigates health care professional (HCP) perceptions of Open Notes and deepens the understanding of the transparency that Open Notes implies.

**Objective:**

Based on two survey studies of HCP perceptions of Open Notes, this paper aims to deepen the academic writing on the type of transparency that is connected to Open Notes.

**Methods:**

HCPs in adult psychiatry in Region Skåne, Sweden, were surveyed before and after implementation of Open Notes. The empirical material presented consists of 1554 free-text answers from two Web surveys. A qualitative content analysis was performed.

**Results:**

The theoretically informed analysis pivots around the following factors connected to transparency: effectiveness; trust; accountability; autonomy and control; confidentiality, privacy, and anonymity; fairness; and legitimacy. The results show that free-text answers can be sorted under these factors as trade-offs with transparency. According to HCPs, trade-offs affect their work, their relationship with patients, and not least, their work tool, the EHR. However, since many HCPs also state that they have not met many patients, and in some cases none, who have read their EHRs, these effects seem to be more connected to the possibility (or threat) of transparency than to the actual effectuated transparency.

**Conclusions:**

The implementation (or reform) of Open Notes is policy driven while demanding real-time transparency on behalf of citizens/patients and not the authorities, which makes this particular form of transparency quite unique and interesting. We have chosen to call it governed individual real-time transparency. The effects of Open Notes may vary between different medical specialties relative to their sensitivity to both total and real-time transparency. When HCPs react by changing their ways of writing notes, Open Notes can affect the efficiency of the work of HCPs and the service itself in a negative manner. HCP reactions are aimed primarily at protecting patients and their relatives as well as their own relationship with the patients and secondly at protecting themselves. Thus, governed individual real-time transparency that provides full transparency of an actual practice in health care may have the intended positive effects but can also result in negative trade-offs between transparency and efficiency of the actual practice. This may imply that full transparency is not always most desirable but that other options can be considered on a scale between none and full transparency.

## Introduction

### Background

One trend in the New Public Management (NPM) movement was an increased demand for transparency [1,2]. The first wave of transparency was connected to performance evaluations and the use of visualizing technologies as a means of regulation, monitoring, and accountability [3-5]. In other words, the transparency implied that policy makers and citizens would gain insight into the work and performance of health care. Stressing the positive effects of transparency, voices were heard advocating for extending its areas of application [6]. As use of the electronic health record (EHR) started to diffuse in Sweden about twenty years ago [7] and about ten years ago internationally [8], a foundation was laid for enhanced transparency within and between health care organizations as well as in relation to their environment [9]. Currently, we appear to be on the next step of this development, as many health care organizations provide patients with online access to their EHRs (here referred to as Open Notes). In other words, transparency increases between the health care organization and the patient. This also implies that a number of different types of transparency are at play simultaneously in the health care systems regarding both what is made visible and to whom.

In the literature, transparency related to visualizing technologies [10,11] and, to a certain degree, the EHR [12,13] has been investigated empirically and discussed in theoretical terms. When it comes to Open Notes, however, most of the research is still at an early stage and either descriptive [14-17] or normative [18,19], with few exceptions [20,21]. Professionals and researchers in the field have interpreted Open Notes as a means of enhancing the transparency of health care professionals’ (HCPs) work and their work tool [13,22-26]. Research on transparency rising from Open Notes reform has been published [27-34], but few attempts have been made to theorize the transparency based on empirical material. Previous research in the area has found that perceptions of Open Notes differ between HCPs and patients, HCPs are more skeptical than patients, and the benefits for patients depend on their condition and ability to take advantage of the technology [35].

Based on two survey studies of HCP perceptions of Open Notes, the aim of this article is to deepen the academic writing on the type of transparency connected to Open Notes. First, because of the gap in the literature indicated above. Second, because the transparency that the Open Notes system permits distinguishes itself from the earlier systems connected to accountability or organizational internal translucence. While Open Notes is advocated to be a part of patient empowerment, meant to engage patients in their health care, the ones that become transparent—the HCPs—are not supposed to be affected. This is different from other types of transparency. In addition, Open Notes has a restricted transparency. In the Open Notes system, the only ones who can view the notes are the patients. These features make the transparency of Open Notes unique and important to study in its own right. The third reason is because the early research has indicated that the noneffect of Open Notes on EHRs as a work tool does not agree with the experience of the HCPs [15]. This gives practical relevance for studying the phenomenon.

We chose to examine perceptions of HCPs when the Open Notes service was implemented in psychiatric care in Sweden. This is because, unlike other parts of health care, psychiatric care has a large degree of vulnerable patients. It is thus likely that some concerns are more salient in the psychiatric context than in other parts of health care where they occur less often because the patients there are considered sufficiently capable of benefitting from the Open Notes service. Previous research has shown that professionals aggregate their concerns before Open Notes is implemented [35]. Thus, we sent out two surveys: one preimplementation and one post. This afforded us the opportunity to compare HCP perceptions before and after implementation and thereby separate belief from experience. In this paper, we analyze the answers to open-ended questions in the two surveys in order to capture the perceptions and deepen the understanding of the transparency that Open Notes implies.

### Transparency in Health Care

Previously, transparency in health care has primarily been mentioned in connection with the NPM wave, where one of the main aims was to shift from what Ouchi [36] calls clan control together with bureaucracies to market control. This in turn led to a demand for visible or transparent processes in health care and quality assurances in communication between the producer and the market. However, production processes in health care have, by tradition, mainly been black boxed and controlled and peer reviewed by the medical professions [37,38]. Thus, the shift to NPM led to reactions and resistance from the professionals [39] because they interpreted the government’s aim for enhanced transparency as being in conflict with their autonomy. According to Levay and Waks [11], efforts to enhance the transparency of professional practice will not only make the practice more visible but will also make it more accessible for external audit and control, which may explain the professionals’ negative reaction to NPM.

The most common academic use of the term or concept transparency in organizational theory comes from the body of literature that views it in terms of its relation to or consequence of governance [40], as in the NPM case. However, the transparency connected to Open Notes does not totally fit into the governance discourse because it does not result in generally open or accessible information for all citizens or for government agencies. In addition, the request for transparency is not aimed at the health care organizations, as such, but at the use of a technology (the Open Notes system) that allows patients to read their health records. The citizen/patient, in turn, can only access his or her own medical information. This can be seen as a transparency on one-to-one basis, that is, between the HCP who writes the note and the patient who reads it. Still, the reform is a consequence of electronic health (eHealth) policies in Sweden where access to more information is expected to empower patients/citizens and increase their participation when it comes to their own health. In this respect, the enhanced transparency can be regarded as a result of governing to some degree. According to Blomgren and Sundén [41], the concept of transparency in this way is often associated with the ideals of democracy, accountability, fairness, informed citizenship, or patient rights.

Transparency has several different definitions and meanings [42]. We have chosen to use Florini’s [43], which states that transparency “refers to the degree to which information is available to outsiders that enables them to have informed voice in decisions and/or to assess the decisions made by insiders.” The definition fits the Open Notes system, the aim of which is to make information available to patients that will enable them to have an informed voice and assess the decisions made by the insiders (the HCPs). Hansen and Flyverbom [44] point out that in the digital age, humans and technologies (materials) together produce transparency through mediating technologies, which also can be recognized in our Open Notes case. Flyverbom et al [45], in turn, state that mediating technologies and the transparency they enable can allow observational control. The authors further propose “that the relationship between power and transparency is best understood in terms of both ‘observational control’ and ‘regularizing control.’” The production of transparency through mediated technologies stands in contrast to the traditional secrecy that Ball [42] states has existed around health records. Thus, the secrecy is not primarily aimed to protect the professionals who write the notes but to protect the patient about whom the notes are written. The Open Notes system has the same aim, in one sense, as the notes are kept secret from all but the patients about whom they are written. At the same time, HCP notes become more transparent than before. This means that secrecy is intact regarding patients but not professionals.

To understand the relationships between different actors involved in the production and use of the Open Notes transparency and the type of transparency it leads to, we were inspired by Heald [46]. Heald constructs an anatomy of transparency considering (1) its direction (upward, downward, inward, and outward), (2) the variety of transparency formulated in three dichotomies (event/process, retrospect/real time, and nominal/effective), and (3) the habitat of transparency.

Heald [46] points out that process transparency may cause efficiency losses both directly (providing the information) and indirectly (more expensive working practices are adopted) and that, in real-time transparency, the “accounting window is always open and surveillance is continuous.” This in turn makes it difficult for the organization (and its members) to focus entirely on its productive activities [46]. Yet another consequence of enhanced transparency is when professionals become more self-monitoring in their everyday work, which in turn can affect them in their core identity [11]. According to Heald [47], there may also be tradeoffs with transparency including “effectiveness; trust; accountability; autonomy and control; confidentiality, privacy, and anonymity; fairness; and legitimacy.” Heald also states that full transparency may not be equal to the most beneficial transparency.

## Methods

### Setting

HCPs in adult psychiatry in Region Skåne were surveyed before and after the implementation of the eHealth service. Adult psychiatry is a part of the Division of Psychiatric Care in Region Skåne in southern Sweden. In 2017, there were more than 575,000 appointments, of which almost one-fifth were with a doctor in the Division, with more than 56,000 unique patients. Patients in adult psychiatry were offered online access to their Open Notes in October 2015, and patients in forensic psychiatry and parents of patients in child and youth psychiatry in Region Skåne were offered the service in February 2019.

### Empirical Materials

The empirical material in this study consists of free-text responses from two Web surveys. The demographics of the respondents, quantitative findings from these surveys, and results from two of the general open-ended questions in the postimplementation survey on how the service influenced patient groups with different diagnoses have been published elsewhere with detailed information about the technical prerequisites of the service, the settings, and the administration of the surveys [14,15]. Thus, the results of these two open-ended questions are not included here. Table 1 presents a summary of information about the two Web surveys.

**Table 1:** Overview of the preimplementation and postimplementation Web surveys.

Aspects	Preimplementation (baseline) survey	Postimplementation survey
Number of questions	34 fixed-choice questions and 3 open-ended questions	44 fixed-choice questions and 20 open-ended questions
Timeframe in which to answer	September 18 to October 2, 2015	March 16 to April 22, 2017
Population	3017 health care professionals	2521 health care professionals
Professional groups included in the population	assistant nurses, doctors, medical secretaries, nurses, occupational therapists, physical therapists, psychologists, and social workers	assistant nurses, doctors, medical secretaries, nurses, occupational therapists, physical therapists, psychologists, social workers, and unit managers
Response rate	28.86% (871/3017)	27.73% (699/2521)
Number of free-text answers	388 free-text answers	1166 free-text answers

The postimplementation survey was based on the baseline survey to permit comparisons between the expectations before implementation and after. Furthermore, both the baseline and postimplementation surveys were based on the surveys developed by the Open Notes Project in the United States [17,48-50]. In both cases, the original surveys were translated and adjusted to fit the Swedish context.

There can be different kinds of open-ended questions in surveys. The most common is the general question where respondents are asked to elaborate on the overall topic of the survey. Another is an expansion question that follows a fixed-choice question [51]. There were 3 open-ended questions in the baseline survey. They were all general in nature and did not relate to any specific fixed-choice question. In the postimplementation survey, there were 20 open-ended questions (the results of 18 of which are included here); 12 were expansions of specific fixed-choice questions in which the respondents were asked to elaborate on the answer given. The remaining 6 open-ended questions were general and did not relate to any specific fixed-choice questions. Both Web surveys were designed so that the respondents could choose not to answer all the questions.

Web surveys make it easy to offer respondents an opportunity to tell the researchers what is on their minds with respect to the survey subject; however, it is still uncommon that free-text answers are systematically coded and analyzed [52]. Free-text answers from open-ended questions in surveys can be described as elicited documents in which the research participants produce the data by answering [53]. One advantage of using this method to gather data for qualitative analysis that Charmaz [53] identifies is that the respondents are able to report things that they, for different reasons, would not have told an interviewer in person. Thus, these elicited texts can reveal sensitive information, and a person can choose to write as much or as little about the subject as they wish. This method for gathering data is appropriate when the participants have a stake in the topic addressed, when they have experience in the area, and when they have the writing skills to express themselves and their views on the topic [53].

### Analysis

The empirical material was analyzed by means of qualitative content analysis. The material was coded by hand in several stages by all three authors. The units of analysis in this study were free-text answers from the baseline and postimplementation surveys. The units of analysis in a qualitative content analysis are identifiable with a low degree of interpretation [54]. When starting the analysis, authors focused on the manifest content of the free-text answers. Manifest content analysis is appropriate when researcher interest is directed at the visible and obvious meaning of the text [54]. Each free-text answer was a meaning unit and there was no need for condensation; the meaning units were statements from the survey respondents with concentrated content in every sentence. Thus, the three authors began to code and then sort out the coded manifest content into categories. A category is on a descriptive level and answers the question “What?” [54]. The research process in this study can be described as abductive, with an initial inductive approach during the coding and categorization of the empirical material from the baseline survey. In the next step, these inductively created categories were used in the analysis of the empirical material from the postimplementation survey. Then a deductive approach was applied during the creation of the final categories that are presented in the results section. An abductive approach is used in a qualitative content analysis when the analysis moves between inductive and deductive approaches during different stages of the research process [55]. The quotes of the respondents used in this paper were translated from Swedish to English by a native English speaking, professional proofreader. They have been slightly edited to improve readability.

## Results

### Overview

The results are structured according to the seven factors described by Heald [47] that can result in trade-offs and synergies with transparency: effectiveness; trust; accountability; autonomy and control; confidentiality, privacy, and anonymity; fairness; and legitimacy. According to Heald, the trade-offs between transparency and these factors are generally positive while there are some conditions where the tradeoffs may become negative. The anticipated effects are briefly described here in connection with each factor. To illustrate the difference between how the professionals anticipated the effects of the Open Notes service before implementation (baseline survey) and how they described their experience one and a half years after (postimplementation survey), we present quotes from the professionals, sorted under before implementation and after implementation. A summarizing analysis is presented at the end of each factor section.

### Effectiveness

The relationship between transparency and effectiveness is mostly seen as positive since public scrutiny is presumed to raise performance. However, transparency that reveals the operational process in an organization may “affect behavior in an unanticipated way,” according to Heald [47]. As shown in the theoretic section of the paper, Open Notes concerns the operational process of the HCPs.

#### Before Implementation

HCPs mainly expressed concerns about how they thought Open Notes would negatively affect the efficiency of their work. They felt that more time would be spent on documentation because they anticipated they would have to change their way of expressing themselves and be less candid in their writing.

*More valuable work time is likely to be spent on record keeping because I will have to be more careful in how I formulate what I write.*

HCPs anticipated that Open Notes would affect the appointment and that the treatment would pivot on the notes in the EHR instead of the patient’s health condition and treatment.

*I see great danger in that the pact with the patient will be broken rather than strengthened, and that much of the work after that will be focused on the notes rather than on the rehabilitation/recovery from the psychiatric diagnosis.*

HCPs felt that when patients were able to read their EHRs online, the contents would change and the health record would not be as efficient a work tool as before for the HCPs themselves.

*I’m worried that we won’t be able to use the health record as the tool it is meant to be.*

But there were also comments about the positive effects that could enhance the efficiency.

*We will gladly go through the notes with the patient. It is valuable for both the patient and coworkers. You can explain and come to a consensus.*

#### After Implementation

One of the most common comments in the postimplementation survey was that the HCPs had not met any (or very few) patients who had read their notes. This was interpreted as lack of efficiency of the service itself and for the patients. This differed from the baseline survey where this had not occurred as an anticipated effect of the Open Notes service.

*Very few patients make use of their notes = very little effect on the patients.*

*Never met a patient who said they read their notes.*

*I’ve only heard from a couple of patients that they have read them.*

In contrast, several comments confirmed experiences supporting loss-of-efficiency concerns found in the baseline survey. HCPs put more effort and time into writing notes in the EHR.

*I weigh my words. How does one describe the manic patient? And the basis of a certificate of illness? Or the young patient with a suspected onset of a psychosis disease?*

*Adds more time to composing each dictation and for all patients.*

*Try to use expressions that are easier to understand which can take a little more time!*

HCPs described how appointments were affected. There was a difference in expression from the baseline study, however, as the quotes were more illustrative, including the patients in the examples.

*When it happens that a patient reads his notes, and, for example, becomes deeply depressed, this is a problem that I have to spend quite a lot of time on sorting it all out. This doesn’t happen often, but when it does, it takes time away from the treatment.*

HCPs also confirmed that the EHR as a work tool had been affected

*The notes become watered down and fall short in their function as a tool for the profession.*

*The notes becomes a poorer quality work document for me as a doctor. I can no longer write down all the tentative diagnoses and hypotheses. Need to simplify the language. Every week.*

HCPs reported their stress levels were raised, affecting their work environment.

*For my own part, there has been an increase in stress to sign off on the notes entries that I’m about 2 months behind on.*

Also, they reported that Open Notes affected the patients’ feelings and perceptions of their illnesses.

*Unfortunately, the patients can get upset when they read their notes, even though they are written respectfully and according to all the existing rules of the art. One patient, for example, suddenly felt worse when she read that she has a chronic risk for suicide. She felt that it meant she was a hopeless case.*

Even though most comments were negative, there were some positive ones about how the service could be used to make communication in the care situation more effective.

*I recommend the notes to the patients as a memory help instead of giving them slips of paper about what we have agreed on. In some cases we agree that I don’t need to send the lab test results as a regular mail letter, but that the patient can instead read about them in her or his notes when I have commented on/assessed the results.*

*I encourage the patient to read often to make sure that I have understood things right.*

#### Effectiveness Analysis

There were three main concerns that the HCPs expressed both before and after the implementation. First, the loss of effectiveness when entering notes into the EHR because they became less candid in the way they wrote. Second, that the appointment was less efficient because it evolved to a larger degree around the notes in the EHR. Third, that the health records became a less efficient work tool for themselves and in communication with other professionals because they were watered down and thus less informative. This makes it clear that the transparency in Open Notes reveals the operational process of the professionals (in Heald’s vocabulary [46]), affects the behavior of HCPs and patients, and impacts the effectiveness of the professionals’ work and the EHR as a work tool. The difference between the baseline and postimplementation surveys is that in the latter, HCPs also described the perceived effects on the patients from reading the EHRs. That, in turn, could affect the care process and make it less effective.

### Trust

Transparency was viewed as being positive in building trust in monetary issues, among others. One of Heald’s examples of a transparency trade-off is that transparency can undermine professional credibility by exposing professional errors (or perceptions thereof) in health care [47].

#### Before Implementation

A main concern of the HCPs was how misunderstandings or dissatisfaction with the content of the EHR would affect the patients’ trust and relationship to caregivers in general and the HCP in particular.

*I am most afraid that a misunderstanding or an unpleasant feeling when reading can, in the blink of an eye, destroy a relationship between the patient and myself that we have built up over several years with some difficult patients. That would be a shame!*

HCPs also suspected this might lead to patients not seeking care even though they need it.

*A great risk that some patients will become distrustful of health care and not seek care again when they really need it.*

But there were also comments that the service can assist in building mutual trust with the patient.

*Psychiatry needs transparency and needs to learn that the patient should be part of the care plan and has the right to information, just as with all other care. This can contribute to the improvement of methods for the staff, and a greater understanding of the patients’ need for transparency and the possibility to influence the care and better cooperation between the staff and patient.*

#### After Implementation

Comments in the postimplementation survey more or less confirmed the concerns that the HCPs had in the baseline survey: patients could react negatively to the content of the EHR notes and this could lead to mistrust toward the caregiver. However, there were more comments about patients’ inability to understand the notes.

*The hardest part is that there are many who do not understand what they read; [they] misunderstand and can feel offended because of that and the way it is written. Many have a need to be understood and be seen. When they then read something that they take to be the opposite, they lose trust.*

Still, some comments indicate that Open Notes could also be used in a positive way to establish trust.

*The best part is to be able to point out that we have a collaboration without a hidden agenda from health care.*

#### Trust Analysis

Comments expressing thoughts (baseline) and experiences (post) of trust or distrust showed that HCPs were concerned about how the patients would interpret the content of their EHRs. In the postimplementation survey, they even expressed doubts about patients’ ability to understand the notes. According to Heald [46], for transparency to be effective, the information that it makes accessible must be understood and used by the recipient. Thus, on the one hand, if the patients do not comprehend the information to the degree that the initiators and implementers of the Open Notes service anticipated, the transparency will not be as effective as planned. The misinterpreted or misunderstood information leads to mistrust between patient and HCPs. On the other hand, if patients do understand and can use the information in communication with their caregiver, trust can be enhanced.

### Accountability

In the public sector, transparency is supposed to have trade-offs with political accountability but also with the distribution of power and resources. Accountability, however, is also about public service providers demonstrating their ability to generate outputs and “process values (such as due process, equity, participation, and deliberation)” [47].

#### Before Implementation

Comments related to accountability mainly evolved around how the HCPs would become accountable to the readers of the notes (the patients) and/or themselves as their notes became more visible.

*I hope that the staff correct inaccuracies if the patient points them out. It is important for the patient that he or she feels that the personnel have understood them correctly. It is about time that the patients gain insight and in so doing, knowledge of the care around them.*

*How should I write the notes so that they meet all the requirements for the stakeholders? It is a work tool and is intended to ensure that relevant information is transferred to those who are involved in care. It must be able to hold up under a Health and Social Care Inspectorate examination. It has to meet my own professional requirements for accuracy and relevant content. And the patients have to be able to be read what I write without causing them harm! I have concerns that the notes will end up being vague and watered down. As a work tool, the notes would be considerably degraded, and that is nevertheless their foremost function! This worries me a lot!*

Positive effects were expected.

*The positive thing is that the staff will become more aware to always document correctly, clearly, and respectfully, and that is very important.*

#### After Implementation

Comments clearly showed HCP concerns had been proven right. They thought about how they were perceived by the readers of their notes in the EHR and how they became more accountable for the writing of their notes.

*The hardest part is the concern of being misunderstood when writing the notes; that my documentation becomes an obstacle in treatment and that I can no longer use it as a work tool.*

#### Accountability Analysis

It was obvious that HCPs, in both baseline and postimplementation surveys, were aware errors could exist in the EHR notes and patients could discover them. This was perceived as both positive, when patients could correct the errors, and negative, since the notes then could be perceived as output that was less valuable or accountable. HCPs also stated they had to be accountable to different stakeholders and it was difficult to know whom to please the most, since the different stakeholders (patients, oneself as a professional, other professionals) may have different needs and/or standards for what is accountable. HCPs also had to follow the laws and regulations surrounding the EHR.

### Autonomy and Control

In Heald’s [47] description, trade-off between transparency and autonomy and control is depicted on an organizational level. Transparency is viewed as an external force that blurs the boundaries of the organization. Since the Open Notes service, however, operates on a one-to-one basis between HCP and patient, we chose to interpret autonomy and control on the individual level—that is, on the HCP level in relation to the external control that transparency toward the patient implies as well as the professionals’ control over their work tool and autonomy over how to write their notes.

#### Before Implementation

Some comments in the baseline study indicated that HCPs were worried about losing control over their work tool (ie, the EHR) when the patients were given online access to it.

*I’m worried that we won’t be able to use the notes as the work tool it is meant to be.*

#### After Implementation

Comments in the postimplementation survey are in line with HCP concerns in the baseline survey about loss of control over their work tool, autonomy over what they can write in their notes, and control over when the information/notes in the EHR should be made visible and to whom.

*I wish there was a function where we who do the documentation could decide when a note becomes visible in the Open Notes; that we ourselves had to approve it.*

*Make sure that an inpatient on a unit cannot block his/her notes from the staff so that the only way medicine dispensation can be registered, for example, is by a staff member overriding the block each time.*

There were concerns about the effect this had on their work process.

*It has become more difficult to write your assessment, as our patients tend to be quick to take offense. That’s why the notes are censured.*

But HCPs also mentioned that the service can give patients more control over their care (or even a false feeling of control).

*Increase transparency and hopefully increase the ability to influence one’s care plan.*

*Create a false sense of control and overview of self [referring to the patient].*

#### Autonomy and Control Analysis

In both surveys, HCPs expressed their worries/experiences of loss of autonomy in how to write notes and control over their work tool. In the postimplementation survey, comments also expressed HCP frustration about not being able to control what information is visible for the patient and when it becomes visible. HCPs thus opposed their inability to control and alter the technical features of the Open Notes service. This also included the fact that patients could block their notes from the HCPs, which of course would complicate the work of the HCPs. On the positive side, there was hope that patients could use the EHRs to more actively participate in and gain better control over their own health/treatment. However, HCPs did not have much experience in their patients making use of this opportunity. It appears that HCP feelings of loss of autonomy and control were not matched by the expected gain in autonomy and control of the patients.

### Confidentiality, Privacy, and Anonymity

Even this set of factors needs to be reinterpreted from the government level, described by Heald [46], to fit the Open Notes. Here, confidentiality is interpreted as being related to the content of the notes since they are confidential as is all information about the patient. The privacy aspect is very closely related to confidentiality, but here the patients can both be forced to and/or break their privacy themselves. Anonymity, however, relates mostly to HCPs and third parties and whether they can be anonymous toward the patient.

#### Before Implementation

When it comes to confidentiality, privacy, and anonymity, HCPs had concerns about patients that could be forced by their relatives or partners to share their EHRs. This would be a type of violation of the patient’s privacy but would also impair the confidentiality between the HCP and the patient.

*The patients who live with threats in close relationships will have a hard time taking the risk of telling how things really are; won’t dare to name or say who is abusing them because that person can pressure them into giving up the password to their notes.*

Other actors, such as an employer, might also insist on reading the information.

*Great risk for privacy violations when notes not only can be read by the patient, but also spread within and outside of the health care system to people who can misuse the information. For example, an employer who knows his/her employee has been admitted asks to read the patient’s notes.*

Professionals’ own needs for anonymity toward the patient were also considered.

*I think there can be an increase in threats and abuse of staff when the name of the staff member who has written the note is included.*

#### After Implementation

Similarities and differences exist between baseline and postimplementation survey comments. There were still comments about the violation of patients’ privacy.

*Those of my patients who said they read their notes are not positive about being able to. They fear that unauthorized people will be able to read about them. One of the patients talks about it at every visit.*

*There still is a little but difficult risk group of those who live with partners in a relationship where violence occurs.*

Concerns about HCP anonymity were reinforced.

*The level of threat has grown worse and personal safety has deteriorated. I think it would have been better if it just said, for example, Nurse Marta in the part that patients can read. If you have an unusual last name, the patient can easily find your home address and that doesn’t feel safe. Patients with addictions sometimes place impossible demands that cannot be met and even have contacts [on the outside] that they can activate to threaten or harm the staff.*

A new area that surfaced in the comments concerned third parties, such as partners or relatives, who share information about the patients that should be kept confidential from the patient.

*I’m less candid about the information that relatives provide. That has sometimes been a big problem because it is important information that otherwise falls out of the system.*

Another new area was information about actions taken that needed to be kept confidential from the patient.

*Patients in outpatient care who are acutely deteriorating and where we in health care, for example, send a notification of concern to social services or in some cases write an institutional psychiatric care certificate that calls for police assistance, where the patients can immediately read this in the notes at home and know that the police are on their way. That poses a lot of problems for us in outpatient care.*

#### Confidentiality, Privacy, and Anonymity Analysis

HCPs were concerned about the loss of patient privacy in terms of their relatives in both surveys. This would also result in loss of confidentiality between HCPs and patients since HCPs cannot guarantee the confidentiality of information given by the patients after the implementation of the Open Notes service. HCPs also expressed fear for the loss of anonymity for their own sake, since their full names would be exposed in the note. There is a difference between the baseline and postimplementation surveys, however, in that HCPs expressed concerns in the latter that third parties could be harmed since the patients’ relatives could not be kept anonymous in Open Notes. They also feared that a patient might gain access to information about actions taken toward them by the police, information that should be kept confidential from the patient but was important for HCPs. HCPs thus expressed both a need to protect patients’ privacy and confidentiality and the anonymity of third parties and confidentiality about information that ought to be withheld from the patient.

### Fairness

Heald [47] points out that in the political debate “fairness is often taken to mean less inequality” but that fairness can also “be conceptualized in terms of rights, deserts, or needs.” In the latter, transparency can invoke envy but in the former, it can stimulate actions to be taken against inequality.

#### Before Implementation

When it comes to fairness, HCPs mainly feared that patients would not have the same access to the service or no access at all.

*Unfortunately, I think that those of our patients who have a computer (few patients) and succeed in getting in and looking (even fewer) will perhaps be more upset after reading their notes.*

Or that if they did read their EHRs, they would not be able to understand them.

*Lack of knowledge and ability to understand the content in the notes can result in increased worry and unnecessary anxiety. Is it ethically right to leave the patient on his or her own to try and interpret the meaning of the notes?*

#### After Implementation

Postimplementation survey comments indicated concern that few patients have the competence and/or resources to access their EHRs online but also that these abilities differed between patient groups (with different diagnoses).

*In the spirit of equality. Several patients don’t have access to or the ability to read Open Notes.*

At the same time, HCPs stated that patients in adult psychiatry, just like those in nonpsychiatric care, should have access to their EHRs through the internet. It is a question of fairness.

*Psychiatry is equated with somatic disorders and access to notes is part of removing the stigma.*

#### Fairness Analysis

One of the arguments for implementing Open Notes in psychiatry was that adult psychiatry patients should have access to their EHRs to achieve equality between psychiatric and nonpsychiatric patients. This was also seen as positive in the HCP comments. Still, the comments in the baseline survey displayed a distrust from HCPs in the patients’ ability to understand and make use of the information in their EHRs. In the postimplementation survey, HCPs, in addition to the above, pointed out that many of the psychiatric patients did not have the material resources to access their EHRs because they may lack computers, the necessary identification to log in to the service, and/or the cognitive ability to understand and manage the information it provides. There were thus both positive and negative connotations related to equality or fairness compared to patients in nonpsychiatric care and even between patients with different diagnoses within psychiatric care.

### Legitimacy

According to Heald [47], transparency can legitimize certain institutions, organizations, or their actions. Here we interpret legitimacy as that of the implementation of the Open Notes service and the service itself in the eyes of HCPs.

#### Before Implementation

Before implementation, comments were about the service itself and its implementation and were positive and negative.

*Poor basis for the decision. Why should we do this? How does it make health care more user controlled/patient safe? Who are the notes for? Should the notes be a work tool between professionals where working hypotheses can be written with the knowledge that those who read the notes have been trained in the language that is used and the content in general? Or should the notes be a diary for the patients?*

*I think it is very good and will use it as an opportunity.*

#### After Implementation

Postimplementation comments were similar to those in the baseline survey. They focused on the implementation and the service itself, why it would not suit the adult psychiatry practice, and its effects.

*A system that was introduced without at all taking into account what the employees in psychiatry think and that only harms the patient and makes it harder to do a good job.*

#### Legitimacy Analysis

The legitimacy of Open Notes was questioned by HCPs on the grounds that the implementation was not sufficiently prepared, other measures were more necessary, and the enhanced transparency negatively affected the professionals’ work tool (the EHR). For some, however, the service also was viewed as offering possibilities.

## Discussion

### Principal Findings

As we have shown, HCP comments in the free-text answers in both the baseline and postimplementation surveys can easily be sorted under Heald’s different factors as trade-offs with transparency. This is not surprising, since transparency toward the patient is the core value in the Open Notes service. The aim of the service is to enhance patients’ empowerment over and participation in their own health care by giving them access to their medical information. In the terms of Blomgren and Sundén [41], the concept of transparency is associated with a number of ideals: democracy, accountability, fairness, informed citizenship, and patient rights. According to the definition of transparency by Florini [43], it increases patient access to information to enable the patient to be informed about decisions made by HCPs and take an informed part in them. While we agree with the definitions above, we would like to contribute to specifying the type of direct, process transparency that the material reveals because it differs from most of the examples of transparency illustrated in theory. The fact that implementation (or reform) of Open Notes is policy driven while demanding real-time transparency on behalf of the citizens/patients and not the authorities makes this particular form of transparency quite unique and interesting. We have chosen to call it *governed individual real-time transparency*.

Since patients have been able to obtain paper copies of their health records on request for decades, the Open Notes service is viewed by policy makers and implementers as a simplifier that has made EHRs much more accessible to patients in both time (whenever) and space (from wherever). The service was not intended to affect the work of HCPs to any degree. However, comments in the free-text answers show that the transparency of Open Notes is a much more complex matter than anticipated by the initiators. The fact that EHRs can be accessed by patients as soon as the HCPs push the enter button changes the type of transparency from transparency in retrospect to transparency in real time, according to Heald’s anatomy of transparency [46]. This implies that the “accountability window is always open and surveillance is continuous,” which does not give the HCP any time to focus on writing the notes without considering that they can be read at any time. This also implies that the transparency that Open Notes allows is what Flyverbom et al [45] describe as observational control that reallocates power between HCPs and patients.

The above described complexity of the transparency of Open Notes may be the main reason for the results from the open-ended questions in the two surveys, which reveal that HCPs experience trade-offs in all seven factors that Heald [47] addresses in his discussion about how enhanced transparency may affect practice. According to HCPs, trade-offs affect their work, their relationship with patients, and not least, their work tool, the EHR. This is in line with Hansen and Flyverbom’s [44] thesis that transparency may add to the problem instead of solving it. However, since many of HCPs state that they have not met many, or in some cases any, patients who have read their EHRs, these effects seem to be more connected to the possibility (or threat) of transparency than the actual effectuated transparency. The fact that patients are able to read their EHRs is key to the HCP reactions rather than whether the patients actually do so. The professionals’ response to Open Notes by changing the way they write their notes is thus a preventive action to a certain extent.

The professionals’ claim that they mainly change their way of writing entries in the EHR system by becoming less candid or leaving out information could in Ball’s [42] terms be interpreted as a way of keeping some of the secrecy intact around EHRs. The HCP comments also confirm Ball’s findings that secrecy toward the patients is primarily aimed at protecting the patients and their relatives. Many HCPs claim that they produce a less complete account of their work and the patients’ medical information with the Open Notes system than they did without it. This means that in some sense, the effects of Open Notes in psychiatry may be that it offers more direct transparency into the EHR but an EHR with less information than before the service. In the terms of Flyverbom et al [45], this can be interpreted as a self-regulatory response to the observational control of Open Notes transparency. HCPs also point out that an EHR that withholds information is less useful to them and their colleagues in their daily work. This confirms the hypothesis by Verheij et al [56] that patients’ access to the EHR will affect the quality of information in the EHR and make it less useful to the following HCPs [56]. This is also in line with Heald’s [47] question whether full transparency is always the best and whether it would be more rewarding and effective for both HCPs and patients if certain sensitive parts of the EHR had been visible to HCPs only.

The comments in both surveys indicate that HCPs change their writing to minimize what they believe are negative trade-offs of enhanced transparency. These trade-offs, as shown above, include loss of autonomy and control over the EHR; impaired confidentiality, privacy and integrity for HCPs, patients, and relatives; and loss of trust between HCPs and patients and more. The total loss of effectiveness in the work of HCPs, both when writing notes and in the care appointment/therapy, can be seen here as the effect of all other trade-offs put together. Postsurvey comments also indicate that many of patients cannot make use of the service, for reasons such as their mental condition, lack of equipment, or their social situation. The authorities’ idea of the rational and capable patient making use of the enhanced transparency offered to them does thus not coincide with the perceptions HCPs have of many of their patients in psychiatry. This indicates that social justice may be hard to reach through governed individual real-time transparency because the possibility that patients can make use of the intended empowerment will always depend on individual situations regarding socioeconomic factors, level of education, interest, and health literacy. This implies a different conceptualization of transparency than the positive connotation it often has had in earlier research.

On the positive side, Open Notes has led to greater understanding and participation for some patients, according to some HCPs. The discourse of increased participation was more salient after implementation than before, indicating that this is an experience rather than a hope. This is in line with the political ambitions of the Open Notes reform and, for these patients, the aim of the reform may well be fulfilled. However, it has not been our intention to decide whether the Open Notes service (reform) has been successful but to broaden the understanding of how enhanced transparency into health care practice can affect HCPs and their work.

### Limitations

This study has several limitations. First, we had no way of knowing if the same individuals answered the questions in the baseline survey and the postimplementation survey. Thus, it is only possible to compare the results from the two surveys on a group level; we do not know if and how individual employees have changed their perception about Open Notes. Second, the response rate was 28.86% to the baseline survey and 27.73% to the postimplementation survey. However, the aim of this article was not to generalize the results but rather to present a deeper understanding of the phenomenon by conducting a qualitative content analysis of all 1554 free-text answers from the two surveys. Third, as mentioned above, we have chosen to study perceptions of HCPs when Open Notes was implemented in psychiatric care because this medical specialty has a large percentage of vulnerable patients. It is therefore likely that concerns that in other parts of health care are less visible because the patients are thought to be capable enough to benefit from the Open Notes service are more salient in this context. Further research is needed to determine whether the results from this study are transferable to other medical specialties. Finally, the baseline and postimplementation surveys were designed in different ways, resulting in a different number of open-ended questions in the two surveys. To permit comparisons between expectations before implementation and experiences after, the postimplementation survey was based on the baseline survey [15]. However, in order to be able to capture the experiences of HCPs in the postimplementation survey, fixed-choice questions and open-ended questions were added. This methodological choice resulted in a different number of free-text answers in each survey. This was not considered to be an issue, however, as our aim was to do a qualitative content analysis [54]. We were not interested in counting frequencies or proportions of similar statements but rather in gaining a deeper understanding of the phenomenon.

### Conclusion

The effects of Open Notes may well vary between different medical specialties relative to their sensitivity to both total and real-time transparency. Psychiatry has been viewed as a particularly vulnerable area when it comes to sensitivity to this type of transparency. This is confirmed by our results concerning both the content of the EHR and patients’ ability to take advantage of the opportunities provided by the service. The results also show that if HCPs react by changing their way of writing notes, becoming less candid in their writing and/or omitting information, Open Notes can affect the efficiency of the work of HCPs and the service itself in a negative way. Additionally, we conclude that HCP reactions are aimed primarily at protecting patients and their relatives as well as their own relationship with the patients and secondly at protecting themselves.

In this paper, we have shown that governed individual real-time transparency that provides full transparency of an actual practice in health care may have the intended positive effects but can also result in negative trade-offs between transparency and the efficiency of the actual practice. This implies that full transparency is not always the most desirable and other options may be considered on the scale between none and full transparency.

## Abbreviations

eHealth: electronic health
EHR: electronic health record
HCP: health care professional
NPM: New Public Management
